# Ambivalent connections: a qualitative study of the care experiences of non-psychotic chronic patients who are perceived as 'difficult' by professionals

**DOI:** 10.1186/1471-244X-10-96

**Published:** 2010-11-24

**Authors:** Bauke Koekkoek, Berno van Meijel, Joyce van Ommen, Renske Pennings, Ad Kaasenbrood, Giel Hutschemaekers, Aart Schene

**Affiliations:** 1 ProPersona Mental Health Care, Pro Persona Centre for Education and Science, Wolfheze 2, 6874 BE Wolfheze, The Netherlands; 2 Altrecht Mental Health Care, Griffensteijnselaan 202, 3704 GA Zeist, The Netherlands; 3 InHolland University for Applied Sciences, Mental Health Nursing Research Group, De Boelelaan 1109, 1081 HV Amsterdam, The Netherlands; 4 Centrum Maliebaan Addiction Care, Tolsteegsingel 2a, 3582 AC Utrecht, The Netherlands; 5 Academic Centre of Social Sciences, Radboud University, Montessorilaan 10, 6525 HR, Nijmegen, The Netherlands; 6 Department of Psychiatry, Academic Medical Centre, University of Amsterdam, Meibergdreef 9, 1105 AZ, Amsterdam, The Netherlands

## Abstract

**Background:**

Little is known about the perspectives of psychiatric patients who are perceived as 'difficult' by clinicians. The aim of this paper is to improve understanding of the connections between patients and professionals from patients' point of view.

**Methods:**

A Grounded Theory study using interviews with 21 patients from 12 outpatient departments of three mental health care facilities.

**Results:**

Patients reported on their own difficult behaviours and their difficulties with clinicians and services. Explanations varied but could be summarized as a perceived lack of recognition. Recognition referred to being seen as a patient and a person - not just as completely 'ill' or as completely 'healthy'. Also, we found that patients and professionals have very different expectations of one another, which may culminate in a difficult or ambivalent connection. In order to explicate patient's expectations, the patient-clinician contact was described by a stage model that differentiates between three stages of contact development, and three stages of substantial treatment. According to patients, in each stage there is a therapeutic window of optimal clinician behaviour and two wider spaces below and above that may be qualified as 'toxic' behaviour. Possible changes in clinicians' responses to 'difficult' patients were described using this model.

**Conclusions:**

The incongruence of patients' and professionals' expectations may result in power struggles that may make professionals perceive patients as 'difficult'. Explication of mutual expectations may be useful in such cases. The presented model gives some directions to clinicians how to do this.

## Background

Across all healthcare settings, clinicians perceive particular patients as 'difficult' [1]. High users of medical services, these patients are generally unsatisfied with the care they receive [2-6] and may evoke strong negative emotions in clinicians [1,7]. Although clearly a subjective and imprecise term, the perception of patients as 'difficult' may result in worse care for patients involved [8,9] and increased stress and burn-out among professionals [10,11]. In the scarce empiric research into patients perceived as difficult in psychiatric services, incidence varies between 6 and 28%[12,13]. Earlier, we found that especially patients who do not comply with the obligations of the sick role as defined by sociologist Parsons [14], run the risk to be perceived as 'difficult' [6]. People have the right to be relieved from their routine social obligations and not be held accountable for their illness, if only they seek and accept professional help, and do their utmost best to restore good health as soon as possible [14].

Among patients perceived as 'difficult', patients with long-term non-psychotic disorders may be seen as not complying with the latter obligation. Unlike patients with psychotic disorders - who are more obviously out of contact with reality - they may be held accountable for their behaviours [6]. Among long-term non-psychotic patients, no particular psychiatric diagnosis is associated with difficulty, while the number of psychosocial problems, psychiatric service use, and ways in which clinicians perceive these patients are [13]. Clinician variables, such as a dominant focus on medical problems over interest in psychosocial issues, however, repeatedly have been found to be associated with perceived difficulty [2-4,13], clearly showing that 'difficult' is defined within the relationship of patient and clinician.

Although substantial research into the patient-clinician alliance has taken place [15], the perspectives of patients in general and long-term non-psychotic patients in particular have hardly been explored [16]. Also we are aware of only one (small) study that explored the care experiences of 'difficult' patients [17]. Here, we focussed on the alliance between the perceivedly 'difficult' patient and the clinician with the purpose to understand why certain patients - according to their accounts of receiving care - come to be perceived as 'difficult'. Thus, we hoped to shed a different light on the labelling of patients as 'difficult' and the possibly poor patient-clinician interactions resulting from it. We stated three research questions: (1) which difficulties do patients who are perceived as 'difficult' experience in their contact with psychiatric clinicians, (2) which explanations do they have for these difficulties, and (3) what changes should be made to decrease these difficulties?

## Methods

### Design

To answer the research questions we used a qualitative Grounded Theory [18] research design with individual interviews of long-term non-psychotic patients perceived as 'difficult' by clinicians. Grounded Theory is a qualitative research method developed for social scientific research, that aims to develop theory grounded in empirical data. It is also widely used in health sciences, mostly - like other qualitative methods - in areas in which current (theoretical) knowledge is limited. Grounded Theory is considered particularly useful in the study of roles and interpersonal processes due to its origin in symbolic interactionism [19].

### Participants

We included patients in public psychiatric care meeting the following requirements, based on a widely accepted definition of severe mental disorder [20]: (1) being in psychiatric care for at least two years, (2) having high psychiatric symptomatology and low social functioning (Global Assessment of Functioning [GAF] score ≤50 [21]), (3) suffering from a non-psychotic disorder on DSM Axis I and/or a personality disorder on DSM Axis II. One subjective criterion regarding difficulty as perceived by treating clinicians was added. Participants had to have had disagreement over form or content of treatment with two or more professionals at least once in the past two years, as assessed by at least two clinicians. A similar criterion has been used in earlier studies [e.g. [12]] and, as imperfect as it is, adds concretization (disagreement), quantity (at least once in past two years), and intersubjectivity (two clinicians).

### Procedure

We selected 12 outpatient departments in three mental health institutes in The Netherlands, striving for a differentiated sample of locations, according to degree of treatment specialization, nature and severity of psychopathology, and geographical dispersion. Key figures of these departments were informed about the research project and were asked to invite clinicians to participate. Treating clinicians (community psychiatric nurses, psychiatrists, psychologists, and social workers) introduced the research to eligible patients as an investigation into difficult relations between psychiatric patients and clinicians. After patients gave consent to establish contact, the first author checked their eligibility with the clinician and then called or e-mailed the patients to arrange an individual interview at their preferred location. After getting acquainted and having explained the project, informed consent, basic socio-demographic and clinical data were obtained prior to the interview. Each participant received a gift certificate to the equivalent of €35/£30.

### Data collection

Two experienced qualitative researchers (BK & JvO) carried out open-ended interviews between March 2008 and September 2009. The research team (BK, JvO, RP, BvM, AK) spent two instructional meetings to immerse in the subject, to design the interview structure and to practice its application. A topic guide, based on a literature search of relevant databases and patient literature was flexibly used [additional file 1]. In the first series of eight interviews, participants were asked after certain topics if they had not mentioned them at all. In the following series of interviews, these checking questions were replaced by questions originating from the analysis of previous interviews.

Participants were invited to start their account by the general question: "Which problems do you experience in contact with psychiatric clinicians, both now and in the past?". Next, the interviewers invited participants to tell in detail about each of these problems and suggest possible explanations for them. Patients were also invited to suggest solutions or alternatives for the present care. All interviews were electronically recorded and transcribed verbatim. Transcripts were analysed in their original language, Dutch, while relevant quotations were translated into English for this paper.

### Data analysis

Data analysis took place between March 2008 and October 2009 in an iterative process, typical to the Grounded Theory-method of constant comparison [18]. Each member of the research team independently coded two out of the first four interviews and checked it against coding by the others [22]. This procedure was followed to construct a mutually agreed on initial code tree, from which further coding could be done by one person (BK), using MAXQDA-software [23].

The research team met after respectively 4, 8, 11, 14 and 21 interviews to discuss progress, monitor interviewers' techniques and congruence, evaluate and conceptually analyze coded interviews, select and explore emerging categories and the mutual relationships, and design theoretical sampling strategies for following interviews. After eight interviews, six main large descriptive categories were constructed to order data. Each category fell apart in three to seven sub-categories. After 11 interviews, a tentative theoretical model of the care process was constructed and a preliminary core category ('incongruous expectations and perceptions of needs') was identified. After 14 interviews, an extensive thick description of data was written, structured according to the six descriptive categories. It was discussed and commented on in the research team, resulting in a number of additional questions used in the following interviews to clarify, refine, and expand the categories. Also after 14 interviews, intermediate results were sent to the participants interviewed for a member check, and were accepted as they were. In addition to the existing questions, in interviews 15 through 21 the tentative model was presented to participants and their feedback was elicited. A summary of the research findings and the final theoretical model was discussed in the final meeting after 21 interviews. Methods and results were discussed with external supervisors (AS & GH) after 8, 14 and 21 interviews.

An example of the analytical process is the *in vivo *(1^st ^order) code 'clinician feels offended', that was categorized under 'clinicians' accountability', then under 'clinicians' professional characteristics', that finally became part of one of the six main categories 'professionals'. Furthermore, because of the both personal and professional qualities of this characteristic of clinicians which was believed relevant to further analysis, a memo (called 'mixing up of personal and professional characteristics') was added to this fragment. Next, other clinician characteristics were explored and coded in detail, paying attention to for instance causes and consequences (*axial coding*). When clinicians' characteristics became part of the central theme of this research, it was further explored in relation to the model later reported on (*selective coding*).

As posited by Lincoln and Guba [24], qualitative research should show sufficient rigour, or 'trustworthiness' in their words. In order to enhance this project's credibility and dependability, member checking was used to validate intermediate findings. Also, peer debriefing was done with the external supervisors, and a thick description was made to allow co-researchers to assess the research' transferability. A detailed log book, consisting of memo's about data collection, analysis, and interpretation, was kept to ensure confirmability.

Ethical approval was obtained from the Institutional Review Board of the organisation the 1^st ^author is affiliated with. Informed consent was obtained from all participating patients.

## Results

In total, 29 patients recruited by clinicians were approached by the researchers. Eight refused (lack of time, lack of interest, or too much stress), 21 were interviewed (duration 26-75 minutes, mean 61 minutes). Almost all participants were socially isolated: living alone, having no (paid) work, having very few meaningful social contacts, and having several psychosocial problems (table 1).

**Table 1:** Characteristics of participants

	n	%
**Age **(mean, sd and range)	38.6 (9.8) [22-60]	
**Gender**		
male	10	47.6
female	11	52.4
**Living arrangement**		
Alone	19	90.5
With partner	2	9.5
Else	0	-
**Housing arrangement**		
Rental	17	81.0
Owned	2	9.5
Living with others	2	9.5
Else	0	-
**Day-time activity**		
Work	2	9.5
Volunteer work	5	23.8
Education/college	0	-
None	14	66.7
Else	0	-
**Number of significant and supportive contacts **(mean, sd, range)	1.7 (1.2) [0-4]	
**Present mental health contact**		
None	1	4.7
Outpatient	18	85.7
Day treatment or inpatient	2	9.5
**Years of mental health contact **(mean, sd, range)	15.2 (7.6) [3-31]	
**Number of psychosocial problem areas **(DSM Axis IV; e.g. family issues, housing or financial problems) (mean, sd, range)	3.2 (2.0) [0-5]	
**Diagnosis**		
*Axis I*		
Chronic depression/dysthymia	5	23.8
Post Traumatic Stress Disorders	5	23.8
Bipolar Disorder II	3	14.3
Attention Deficit Hyperactivity Disorder	1	4.7
Any substance abuse disorder	3	14.3
		
*Axis II*		
Borderline Personality Disorder	12	57.1
Personality Disorder Not Otherwise Specified	7	33.3
		
Axis I only	2	9.5
Axis II only	7	33.3
Both	12	57.1

From the 17^th ^interview we did not collect data that added significantly to our findings. Thus, we carried out four additional interviews (18-21) to ensure that we reached theoretical saturation, and concluded data collection after interview 21. Overall, interviews proceeded relatively smoothly. Some patients expressed substantial grief, anger, or despair about current or past mental health contacts. The interviewers then paused, validated these emotions, and inquired whether the participants wanted to terminate the interview - which did not happen in any instance.

Our qualitative analysis was guided by six large categories of which four referred to actors: patients, clinicians, psychiatric services, and the patient's social system. Two other categories referred to interpersonal processes: contact between patient and professional, and treatment of the patient's problems by the clinician. These six categories are used to structure the answering of the three research questions in the results below, and specifically to construct a model of the patient-professional interaction in the second part of the results-section.

### Difficulties experienced by 'difficult patients'

Almost all participants described themselves as being 'difficult' for professionals, either because they knew they were perceived as such or because they said that they were not 'regular customers'. Participants described (1) challenging behaviours exhibited by themselves towards clinicians and services, (2) difficulties in contact with individual psychiatric clinicians, and (3) difficulties with mental health care services.

Patients described behaviours that could be perceived as 'difficult' in quite some detail. These varied from not showing up on or walking away from appointments, to disqualifying and offending professionals, to shopping around for help, or claiming, threatening, fighting and stalking professionals. With regard to these behaviours, many acknowledged their heightened sensitivity for interpersonal rejection, personal history of problematic relationships, and high expectations of psychiatric services. These services are a last resort for many of them, often related to the absence of substantial social support. Patients' sometimes very outspoken expectations of clinicians and services are, in their view, repeatedly not being met. The following citation exemplifies an expectation that may not be particularly high, but clearly very different from what psychiatric clinicians are able or willing to offer.

*In the beginning I had this ideal picture of day treatment, that they would comfort me and such things. That did not happen though, instead when I laid down on the couch they said that I could not do so. *[P15]

*But you do have a preset expectation (...), like they will start helping me now. You do not think that you will have to do the work, no, you believe they will do it. *[P19]

The expectation 'to be helped' is recurrent in many participants' accounts. Patients feel a strong need for help but actually do not know what can be done. Clinicians in turn, in complex cases, do not know either which tends to culminate in mutual powerlessness.

*Can we do anything else for you, they asked. I don't know, I said. (..). I mean if I all knew so well than I would not be here, would I?? *[P11]

The second kind of difficulties are those regarding interpersonal contact with clinicians, in which participants differentiate between 'personal characteristics' and 'professional characteristics'. On the personal level, participants in particular miss true interest and authenticity. This stretches farther than politeness or professional courtesy, farther than just being listened to. For many participants, clinicians' merely professional interest seems insufficient, possibly related to their aforementioned high expectations. Some participants make a direct link between their own difficult behaviours within the mental health contact and the lack of 'right interest' from clinicians. If there is no such true interest, these participants tend to stay away or start acting in a way that may be perceived as 'difficult'.

*When I say something out of personal experience some doctors reply 'well who has went to school for this?'. Those kind of remarks make me very, very angry. *[P13]

Professional characteristics participants search for in clinicians, are taking the lead, accepting responsibility, and setting out a clear course of treatment. An empathic and understanding attitude does not suffice, participants also want their clinician to assess them correctly, to look beyond their initial presentation and confront their easy excuses. While the aforementioned personal characteristics (true interest and authenticity) are most important to the interpersonal process of contact, clinicians' professional qualities are most important for the treatment process. Participants clearly state that these professional characteristics, however important, come into play only when a good-enough contact with the clinician has developed. At the same time, in many of the participants' accounts, personal and professional characteristics are not so clearly distinguishable. For instance, taking responsibility is not only seen as a strong professional asset but also as a sign of personal involvement, of real interest, and even of warmth.

*They decided to take me by the scruff of the neck and help me. They did not give up on me. And that is what I am enormously grateful for now. *[P2]

In some cases the desire for warmth and responsibility goes as far as one participants wishing for a long-term compulsory admission.

*But for a psychiatric patient, who has no-one, an involuntary admission may mean that there is still one person on the earth, even though it is an institution, that at least cares a bit about my fate. *[P12]

The wish for clinicians' personal involvement, however, is limited by the extent to which clinicians bring their own emotions into the contact. Clinicians' strong emotions are perceived as a source of potential difficulties by participants. For instance, one participant described a therapist that addressed the patient's noticeable alcohol odour due to drinking the night before. She expressed her personal feelings about the patient coming to their first appointment hung over and kept on repeating her discontent.

*She did not ask one single question, all she did was whine about what I had done to her. Yeah, right. Well, now I go home and hang myself - how would *that *make her feel? *[P3]

In line with this, several participants state that clinicians tend to interpret 'difficult' behaviours far too easily as personally directed towards them. They want clinicians to be more neutral in such cases, to understand certain behaviours as part of the patient's disabled behavioural repertoire and to asses it correctly as meaningful or functional. Yet at the same time participants loathe this neutrality when it turns into a distant, objectifying attitude. This puts the professional in a one-up position which many patients find hard to tolerate.

The third kind of difficulties are those with psychiatric services, which tend to hamper access by all kinds of complex organisational procedures, such as low contactibility of clinicians, limitation of care, and high thresholds for certain treatments. Also there are unwritten rules, so they say, considering themes that are apparently not appropriate to discuss or do. These issues are at odds with the involvement participants desire. At a more abstract level, participants note collective negative attitudes in psychiatric clinicians, exemplified by the negation of patients' positive characteristics and pessimism about recovery opportunities. While participants feel that their illness, deviance, and difficulty is focussed on constantly in psychiatric services, they also experience that in order to maintain their contact or to receive treatment, they should behave as 'good' patients (i.e. seek and accept help and do their best to get better as soon as possible).

*Professionals continuously laid demands on me about what I could or should not do. Never positive about what I could or should do. *That *I can draw strength from. Not from demands or expectations of what I should or could not do. *[P15]

Participants state that in psychiatric services, patients' failures and pathology are constantly paid attention to and pointed out. Yet at the same time these pathological behaviours (e.g. using illicit drugs, self-mutilating or attempting suicide) are not tolerated and may be reasons to refer or discharge patients, which may be one of the unwritten rules referred to above.

*I came there and could not smoke marihuana, I could not self-mutilate, I could not... But what I *could *do was unclear to me. I did not understand it. *[P15]

Another participant tells about her admission to a hospital because of suicidal intentions, where she had to hand in her medication. After refusing this, she was discharged (still in possession of the pills).

*That serious they took the problem, they put you back on the street. (...). Try to keep someone inside and to make contact with where someone's at, do not start a struggle over pills or self harm. That I still find so strange that people are put on the street because they do that *[self harm]*. No, I find that cruel, truly cruel. *[P14]

Another such account:

*I grew only more suicidal and destructive. All the time I got some sort of slap in my face: you better leave, we can't do anything for you. All it was, was a confirmation that I did not belong there, that I was nothing. *[P19]

### Explanations for perceived difficulties: lack of recognition

We now move to possible explanations for the difficulties in the patient-clinician relationship. All patients want clinicians to recognize their suffering and their needs. This recognition of needs, however, does not automatically mean that patients want to be seen as *patients in need*. Many find it hard to accept the patient role, or even concur with their given diagnosis. A distant and strictly medical approach (i.e. being offered diagnosis, prognosis and treatment by a skilled doctor) was endorsed by none of the participants. While they believed this to be a necessary but not sufficient element of care, it was once again pointed out that treatment cannot exist without contact. For some, receiving a diagnosis meant recognition of the genuineness of their problems and suffering.

*But if you have an appointment with a psychiatrist who does not say what is best for you than you do not have it. You don't have that little paper that says what is exactly wrong with you. *[P5]

*Well, I was happy that I finally could, well, give it a name. That it was truly something. A personality disorder, or whatever you want to name it. *[P11]

For others, receiving a diagnosis exemplified the inequity of the patient-professional interaction. With personality disorders, participants often resented their given diagnosis since they believed it actually hampered access to health care. Some expressed the wish to receive a diagnosis unburdened with the notion of 'being guilty' of their behaviour, in order to have better access to services. As such, different notions by patients and professionals of both the function and type of diagnosis may be partly explanatory for difficulties.

Independent of diagnosis, all participants expressed a deep need to feel understood, and in some cases, to be cared for by health professionals. The mental health system was described as a far from ideal but still the best environment to have this need met, better than their - so often absent - social system or other helping agencies. In other words, mental health care offers the least bad environment, shown by the statement of a participant who expresses her feeling to be relegated to mental health care.

*People don't understand that *[vulnerability] *at all. It is such a lack of recognition. (...). Then, psychiatry is the lesser of two evils. That is why I stay there, I believe. I do occasionally have a good conversation, or I am sometimes able to find some relief. Otherwise I only start doing crazy things and become more sad. *[P14]

From this point of view we may understand difficulty partly as a consequence of patients' ambivalence towards psychiatric care: needing it without wanting to. This perceived need merits further attention, since in spite of previous negative experiences and expressed discontent with several clinicians' characteristics, participants do remain in psychiatric care.

*It *[psychiatry] *does not bring me any further, it does not offer any grip. It is not something one can pull oneself up on like for instance work is. Once again, I will always keep on going there *[mental health care] *without wanting to. *[P8]

They appear to be looking for exceptions to the rule, for the one clinician that does understand them. Some are able to find this person but many are not and keep on fighting the misunderstanding they experience. Many clinicians appear to be unable to truly identify and validate the needs of these patients. At the same time, these needs may be so existential that psychiatric services will never be able to accommodate them, as exemplified below.

*I expect, and that appears to be undeliverable, my basic problem is that I just want my mother. But that one simple thing is not available in psychiatry. *[P12]

Instead of 'tender loving care', patients get 'distant' advice and structure. Many deeply resent the 'doctor knows best'-attitude of some clinicians, and do not want to be told what their life is, or should be like. Such active, but often also strict and formal clinicians, are easily perceived as bringing about a power imbalance that takes away the patient's control over the treatment encounter, and even the patient's life. Yet, not having to be in control also relieves patients from their obligations and clearly acknowledges their needs and limitations in doing things themselves. Two participants exemplify this paradox in vivid terms.

*These power relations feel very safe on the one hand because you just don't have anything to say anymore. Really, that security from when you were a child. Everything is being done for you and you just have to do this at that time and nothing else really. But on the other hand, it is not good since you cease to be a person. *[P4]

*At the moment I am not right, I feel very dependent, really very small. Then I think, oh no, I really need them. Yet, when I feel better, I am annoyed about them and their idea that they can decide what is good for me *[P13].

Patients once again appear very ambivalent about truly accepting help and the patient role. They express their difficulties with being either a person who is competent and autonomous, or a patient who is incompetent and dependent, and appear unable to combine those. Yet, according to participants not only patients have difficulties relating to this polarized notion of autonomy and helplessness. Clinicians also have difficulties to tolerate these two sides of one person, and tend to respond paradoxically to patients that display either one of them. Whenever a patient appears able to communicate his or her needs clearly, professionals see this as a sign of good mental health. So, when the patient asks for help in a 'normal' way, that is without dramatizing, threatening or without visibly being shattered, clinicians tend to believe that help is not actually required.

*They said: 'you can articulate it so clearly, we believe that nothing is necessary'. That I found so bizarre, since I was doing everything to articulate myself clearly since otherwise I could not bring the message across. I would not receive help when I articulated it poorly, nor when I articulated my needs clearly. *[P11]

Implicit notions about help-seeking behaviour are suggested by these examples. Clinicians expect patients to ask for help in a non-dramatic, rational, but still indigent way. Patients should thus not come up too autonomous or dependent, since clinicians seem to hold unspoken views of what is the right way to ask for help. When the patient is highly autonomous, the clinician appears to be unnecessary and may feel unseen him or herself. When the patient is overly dependent or 'needy', the clinician sees this as overreacting or even manipulative, and as potential risk of dependency. Patients desire a special kind of understanding and compassion from clinicians, that incorporates both their personal qualities and their difficulties, and not solely focuses is on what is wrong, or easily concludes that nothing is wrong. Clinicians, on the other hand, are easily confused over patients' presentations and tend to take adequate help-seeking behaviour for the absence of problems and needs. Margins for both patients' and clinicians' behaviour appear very narrow, which we will further exemplify in the next paragraph.

*I am afraid that it is a mixture of my own paranoia and hostility towards health professionals, and the way I interpret what they say. And the interaction that comes from this. (...). Plus that they have this panic-like fear for dependency of patients. *[P12]

### Changes in patient-clinician contact: using the 'therapeutic window' through different stages

**Figure 1:**
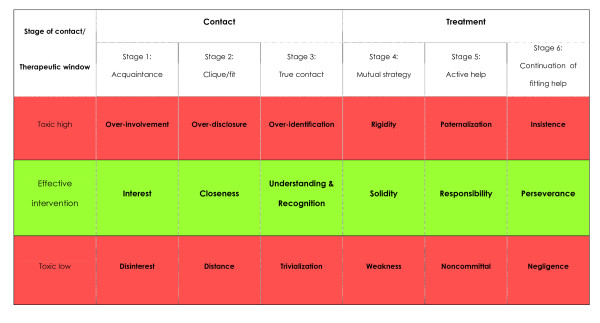
**Stages of contact, interventions, and respective therapeutic windows**.

The narrow margins of 'right' behaviour of both patients and clinicians described above, returned across many interviews and categories. Also, they were not static entities but changed over time. This closely relates to the core category we came to construct: incongruence of expectations and perceptions of needs. Participants repeatedly described wanting something else than professionals: more or another kind of care, more (or less) personal involvement, or a more structured approach to problems. Combining this with another recurring finding, that of contact and treatment as two separate dimensions, we tentatively constructed a stages model in the contact process with 'required' clinician behaviour per stage (figure 1). In each stage, there is a 'therapeutic window' of optimal clinician behaviour, and two wider spaces - both below and above the therapeutic dosage - of 'toxic' behaviour.

The first three stages of this model (figure 1) all concern 'contact', while the latter three concern 'treatment'. In the first stage ('acquaintance') patient and professional meet and get basically acquainted. Patients expect some basic interest of the professional at this stage, while rapid over-involvement or clear disinterest may be toxic and prevent the patient from returning for a next meeting. The next stage ('clique/fit') requires more closeness from the professional, but not over-disclosure of personal information or too much distance. A clique refers to a certain level of personal contact that shows the patient that the clinician cares.

*I think it's a clique, it has to do with a clique. A clique between professional and patient is very important. Because if it cliques, then you gain trust. *[P2]

The third stage ('true contact') is a crucial one, in which the clinician needs to recognize and genuinely understand the patient with both his or her qualities and shortcomings, as well as the patients' suffering. In this stage most difficulties tend to arise, since expectations are up from the previous stages. Patient and clinician must navigate themselves through all the ambivalent demands described in the previous paragraphs. Toxic responses by clinicians include over-identification with the patient, and trivialization of problems and needs since this reinforces patients' earlier experiences of uncaring clinicians. In this stage, toxic clinician behaviour may result in more intense patient responses (e.g. becoming disqualifying, angry, clinging, or threatening) than not returning for another appointment.

*I believe that because when you are recognized, you are heard, and then you don't start fighting all the time to be heard. *[P14]

The second three stages all concern 'treatment'. The fourth stage ('mutual strategy'), is the one in which the content of treatment becomes involved. A mutual agreement over goals and a treatment strategy need to be developed. In order to do this, more than just understanding is required, the clinician needs to be active and directive. This solidity should not be too rigid, or be too weak, since both are toxic to patients that look for a clear course.

*And then the conversations start to dilute into something I can't define any more. (...). Then I have completely lost track. There is no structure any more, no direction. Yeah, at a certain moment, yeah, you just stop going. *[P3]

In the next stage ('active help') the clinician should show not to be afraid to take responsibility for the patient's well-being and show continued involvement. Participants state that it is important that clinicians show their willingness to do some work for their patients. Failure to find a non-toxic level of intervention may result in patients perceiving the clinician as paternalistic or non-committed.

*And if there's some time left, they ask me if they should join me to social services or anything. And that is really great sometimes, because it makes me more motivated to do start doing such things again by myself. *[P9]

In the sixth and final stage ('continuation of fitting help') clinicians must carefully monitor the care process for recurring or new difficulties in the contact. The clinician needs to be perseverant in focussing on treatment goals, and vigilant for possible breaches in the contact. Too much persistence can result in rigid insistence, which like its opposite - negligence - is toxic to the patient.

*So there is little attention for the progress one has made. Is he feeling better, is it right what we are doing here? *[P5]

We may state that the therapeutic window for interventions with 'difficult' patients is very narrow. In each stage things can go wrong due to either the lack of, or the excess of this required behaviour by clinicians. In both cases, such behaviour may be toxic to patients who are in substantial need of recognition of their problems and needs as described before.

## Discussion

This research explored the views of patients perceived as 'difficult' on their contacts with psychiatric clinicians and services, in order to improve our understanding of difficult treatment encounters. We found that patients have difficulties with a variety of clinicians' and services' characteristics, of which disinterest, noncommittal, and a general negative view are the most important. The interpersonal process of perceived lack of recognition, grounded in the incongruence of expectations of one another, may be considered the major explanation for difficulties between patients and professionals. We constructed a staged model in which the development of personal contact is most important to patients during the first three stages, and to which substantial treatment is added in the next three stages. The stage in between personal contact and substantial treatment is pivotal and concerns the recognition of patients as both genuinely ill, and valuable human beings with capacities and shortcomings.

### Substantial findings

Although the starting point of this research, and the premise of our sampling strategy, it cannot be upheld that 'difficult' is an attribution that can be objectively made upon patients. The findings of this study thus deserves interpretation on different levels.

A first important finding on patient level is that perceived difficulty may partly be explained by the ambivalence of these patients to fully assume the patient role. This appears to be a central feature of all participants and explains why such patients are found among people with quite different diagnoses. Not specific diseases themselves, but the way people perceive them and the way they want health clinicians to respond to them, appears associated with difficulty. Also, it explains why these patients evoke such strong and ambivalent emotions in health professionals. If the patient is unwilling to accept the patient role, a clinician cannot take up the designated role of genuine helper. It is quite well established that any health professional whose help is denied, questioned, ridiculed or whatsoever, feels frustrated [e.g. [1,3]]. To a certain extent, the 'difficult' patient who feels unseen, unheard and unrecognized, is mirrored by the clinician who remains unrecognized as a genuine helper.

A second important finding, on professional and services level, is that mental health care does not very well know how to respond to patients that behave different and less predictable than other patients. The response of choice to patients that are ambivalent about being a patient, seems to be an intensification of efforts to make him or her fit the 'normal' patient frame - which in fact has the opposite effect. For instance, assuming the expert role to convince the patient to behave differently, is exactly what will exacerbate the patient's unpreparedness to do so. It may be much more effective for the professional to recognize, voice, and discuss the patient's ambivalence.

A third finding, that encompasses different levels, is that patients who are perceived as 'difficult' and their clinicians who perceive them as such, have very different expectations about the contact with one another. The expectations patients have in different stages of the interaction with health professionals have been exemplified in the model. This model offers insight into the various expectations and allows clinicians to discuss these with patients in different treatment stages. Clinicians may thus use this knowledge to explicate mutual expectations and set up mutually agreed on goals and actions.

### Limitations and strengths

There are limitations to our study. First, the results need careful interpretation since they potentially suffer from a self-serving bias of participants. Very much like clinicians in earlier research [13], patients primarily report behaviours of the other they have trouble with. Second, our findings do not apply to psychiatric patients that are sent, or even sentenced, to mental health care. Third, we were unable to use alternative data sources to verify our findings (triangulation [25]). Despite several invitations, none of the participants was willing to attend a focus group discussion to verify intermediate findings and collect new data. Fourth, sampling proved to be complicated during the entire research for which reason selection bias is a risk. Many clinicians did not readily enrol possible participating patients, notwithstanding the description of this project as research into difficult interactions. Also, the requirements of both purposive sampling (to allow variation of socio-demographic characteristics, psychiatric diagnosis and health care settings) and theoretical sampling (following from intermediate analyses) limited the number of suitable participants. Also, initially enrolled patients did not always follow through when the interview date came closer. The period of data collection was therefore substantially extended. Potential undersampling of the most 'difficult' patients, however, is countered by the fact that participants, who were announced as 'really difficult' patients by clinicians, proved to be willing and even eager to participate. We believe that refusing research cooperation is not a primary characteristic of this population, thus suggesting the absence of selection bias on these grounds. Although our sample size was smaller than intended, theoretical saturation appeared relatively soon, and was followed by four additional interviews to ensure validity. To our knowledge, this is the first qualitative study into the experiences of 'difficult' patients using a sufficient sample size and rigorous qualitative methodology.

### Current and future research

Our findings, and especially the model, concur quite well with, and add some detail to, the literature on the importance of the therapeutic alliance in psychiatric treatment and the required focus on bonds, goals, and tasks [26,27]. The importance of true interest in, and recognition of, the patient and his or her suffering, is under different names also found in modern care models for different non-psychotic disorders [28-30]. More surprisingly, findings from studies of 'difficult' patients with medically unexplained symptoms in general health care, are quite consistent with ours [e.g. [31]]. In this study, patients expectations also differed from those of doctors, while in another study [32] the recognition of suffering, followed by a open discussion of treatment options was a finding comparable to our findings. Future research into difficult alliances may sample pairs of patients (both perceivedly 'difficult' and 'non-difficult') and professionals, both investigating their mutual expectations, interactions, and progress over time.

## Conclusions

The incongruence of some patients' and professionals' expectations may result in power struggles that may make professionals perceive patients as 'difficult'. Explication of mutual expectations may be useful in such cases. Additionally, clinicians may first wholeheartedly acknowledge and recognize the needs of such patients, only to proceed with more formal treatment procedures (such as clarification of expectations, setting of goals, and choosing of interventions) from there. The presented model may be helpful to navigate through the different stages of the patient-professional contact.

## Competing interests

The authors declare that they have no competing interests.

## Authors' contributions

BK, BvM, AS and GH devised the idea of the study and designed the methods. JvO conducted, and RP participated in the interviews. BK, BvM, JvO, RP and AK performed analyses of the data and regularly discussed progress. BK led the data collection, analysis, and prepared the manuscript. BvM, AK, AS and GH co-drafted the manuscript. All had full access to all data. All authors read and approved the final manuscript.

## Pre-publication history

The pre-publication history for this paper can be accessed here:

http://www.biomedcentral.com/1471-244X/10/96/prepub

## Supplementary Material

Additional file 1 **Literature review of documents written by patients**. search strategy and results of a review of patient documents in the psychiatric literature. Click here for file
