# Supplementary material for: Ambivalent connections: a qualitative study of the care experiences of non-psychotic chronic patients who are perceived as 'difficult' by professionals
Source: BMC Psychiatry. 2010 Nov 24;10:96. doi: 10.1186/1471-244X-10-96 (PMC3002895; doi:10.1186/1471-244X-10-96)
Supplement: Additional file 1 — Literature review of documents written by patients. search strategy and results of a review of patient documents in the psychiatric literature. [file 1471-244X-10-96-S1.DOC]

**Supplementary file to manuscript *Ambivalent connections: a qualitative study of the care experiences of non-psychotic chronic patients who are perceived as ‘difficult’ by professionals.***

*Literature review of documents written by patients*

Search strategy:

(1) Systematic search in databases

- "Anecdotes as Topic"[Mesh] AND "Mental Disorders"[Mesh] AND "Professional-Patient Relations"[Mesh] *PubMed: 38*
- “Narratives” AND "Mental Disorders"[Mesh] AND "Professional-Patient Relations"[Mesh] *CINAHL: 43*
- “Narratives” AND “Mental disorders” AND (“Interpersonal communication” OR “Therapeutic Process”) *PsychInfo: 70*

(2) Purposive search in databases/internetsites of general patient organisations

- Literatuurlijst Pandora Foundation (NL)
- Dossier Psy (NL)
- Rethink.org (UK)
- NAMI + affiliated sites (US)
- National Empowerment Centre ([www.power2u.org](http://www.power2u.org/)) (US)

(3) Purposive search in scientific journals

- serie egodocuments in *Psychiatric Services*

(4) Existing dataset at the Gelderse Roos Institute of Professionalization

- patient interviews from 2003

Results (themes):

- wrong/no/unclear diagnosis
- negative treatment based on diagnosis (Axis II) – little differentiation between psychiatric disorders by professionals
- too strict distinction between medical and psychiatric problems
- too strict distinction between substance abuse and psychiatric problems
- lack of information
- blame of secondary gain and attention-seeking
- use of coercion/power
- clinicians riding their hobby horses
- guilt induction by clinicians
- denial of problems/illness
- poor organisation of care
- refusal to offer long-term therapy
- staff turn-over, each one having different ideas about what needs to be done
- passing on of ‘negative’ diagnoses from one clinician to another before having seen the patient
- lack of continuity and accessibility of care
- no involvement of family system
- stupid therapies
- not listening/no clique
- only focus on medication treatment

## Main themes

1. lack of information (about diagnosis, treatment, and alternatives)
2. negative treatment (unkind, disrespectful, arrogant)
3. organisational shortcomings (‘walls’ between departments, no continuity of care)
4. patient is blamed for illness or behaviour (anger about suicide attempt, perceived secondary gain)
5. lack of agreement with clinician (patient want long-term therapy, no medication; clinician want the opposite)
6. lack of interest and true listening
7. no interest in social system /significant others
8. not being taken seriously because of a given psychiatric diagnosis
